# Supplementary material for: Clinical characteristics associated with mortality of COVID-19 patients admitted to an intensive care unit of a tertiary hospital in South Africa
Source: PLoS One. 2022 Dec 30;17(12):e0279565. doi: 10.1371/journal.pone.0279565 (PMC9803161; doi:10.1371/journal.pone.0279565)
Supplement: S1 Table — (DOCX) [file pone.0279565.s001.docx]

**S1 Table A: Frequency distribution of medication used among COVID-19 patients admitted in ICU**

| **Medications used** | **Total number** | **Number (percent)** |
| --- | --- | --- |
| Antibiotics |  |  |
| Amoxicillin/Clavulanic Acid | 401 | 265 (66) |
| Azithromycin | 401 | 257 (64) |
| Meropenem | 401 | 172 (43) |
| Vancomycin | 401 | 86 (21) |
| Colistin | 401 | 41 (10) |
| Corticosteroids |  |  |
| Dexamethasone | 401 | 207 (52) |
| Methylprednisone | 401 | 42 (10) |
| Hydrocortisone | 401 | 86 (21) |
| Antifungal |  |  |
| Fluconazole | 401 | 37 (9) |
| Anticoagulants |  |  |
| Enoxaparin | 401 | 393 (98) |
| Other medications |  |  |
| Vitamin C | 401 | 268 (67) |
| Thiamine | 401 | 368 (92) |
| Proton pump inhibitor (PPI) | 401 | 168 (42) |
| Aspirin | 401 | 168 (42) |
| Spironolactone | 401 | 38 (9) |
| Losartan | 401 | 43 (11) |
| Simvastatin | 401 | 75 (19) |
| Other hypertensive medication | 401 | 184 (46) |
