# Supplementary material for: Clinical characteristics associated with mortality of COVID-19 patients admitted to an intensive care unit of a tertiary hospital in South Africa
Source: PLoS One. 2022 Dec 30;17(12):e0279565. doi: 10.1371/journal.pone.0279565 (PMC9803161; doi:10.1371/journal.pone.0279565)
Supplement: S2 Table — (DOCX) [file pone.0279565.s002.docx]

**S2 Table B: Frequency distribution of laboratory parameters at initial measurements among COVID-19 patients admitted in ICU**

| **Laboratory Parameters** | **Total number** | **Median**  **(Number)** | **IQR**  **(Percent)** |
| --- | --- | --- | --- |
| Sodium (Na) | 405 | 137 | 134-140 |
| Creatinine | 405 | 77 | 63-107 |
| Potassium (K) | 405 | 4.3 | 3.9-4.9 |
| Chloride (Cl) | 405 | 98 | 95-102 |
| Haemoglobin (Hb) | 405 | 13.1 | 11.8-14 |
| Haematocrit | 405 | 0.4 | 0.361-0.433 |
| Platelets (Plt) | 405 | 294.5 | 221-376 |
| Urea | 405 | 6.4 | 4.7-9.2 |
| White Cell Count (WCC) | 405 | 11.27 | 8.3-14.32 |
| Lymphocytes | 404 | 0.94 | 0.63-1.28 |
| Neutrophils | 404 | 9.38 | 6.89-12.36 |
| C-Reactive Protein (CRP) | 400 | 184 | 118.5-282.5 |
| Procalcitonin (PCT) | 399 | 0.44 | 0.19-1.09 |
| D-Dimer | 395 | 1.05 | 0.46-4.40 |
| Ferritin | 362 | 1072 | 642-1702 |
| INR | 362 | 1.125 | 1.05-1.22 |
| PTT | 107 | 25.9 | 23.1-30.5 |
| NT-proBNP | 367 | 354 | 100-1223 |
| Troponin T | 361 | 14 | 9-33 |
| Albumin | 83 | 30 | 26-34 |
| Alanine aminotransferase (ALT) | 388 | 31 | 20-50.5 |
| Aspartate aminotransferase (AST) | 207 | 49 | 34-72 |
| Alkaline Phosphatase (ALP) | 100 | 91.5 | 69.5-119.5 |
| Gamma-glutamyltransferase (GGT) | 79 | 89 | 45-164 |
| Calcium | 253 | 2.05 | 1.97-2.16 |
| Magnesium | 250 | 0.92 | 0.82-1.05 |
| Phosphate | 246 | 1 | 0.8-1.21 |
| HbA1c | 302 | 6.7 | 6.2-9.3 |
| Total Bilirubin | 390 | 7 | 5-10 |
| GFR^^[[1]](#footnote-1)^^ (not normal, %) | 381 | 261 | 68.5 |

1. Non normal (GFR<=60) and normal when GFR >60. [↑](#footnote-ref-1)
