# Supplementary material for: Clinical characteristics associated with mortality of COVID-19 patients admitted to an intensive care unit of a tertiary hospital in South Africa
Source: PLoS One. 2022 Dec 30;17(12):e0279565. doi: 10.1371/journal.pone.0279565 (PMC9803161; doi:10.1371/journal.pone.0279565)
Supplement: Attachment — Submitted filename: Reviewers responses_25 May 2022.docx [file pone.0279565.s003.docx]

**Responses to reviewers’ feedback	24^th^ May 2022**

Thank you for sending us the comprehensive feedback. We are grateful to you for this wonderful feedback, it has helped us to improve the quality of the manuscript and turn it a more informative piece of work for the readers. The manuscript entitled: “Clinical characteristics associated with mortality of COVID-19 patients admitted to an Intensive Care Unit of a tertiary hospital in South Africa” has been revised accordingly.

**Reviewer #1:**

In this study Nyasulu et al describe Clinical characteristics associated with mortality of COVID-19 patients admitted to an Intensive Care Unit of a tertiary hospital in South Africa. The authors conclude that the mortality rate in COVID-19 patients
admitted to the ICU was high and that older age, the need for invasive mechanical ventilation, HIV status, and metabolic acidosis were significant predictors of mortality. Overall, the manuscript is of potential interest. My comments are as follows:

**Comment 1:** My major comment is that there is too much focus on discussing possible associations that did not remain significant in adjusted analysis. The authors should revise results and discussion appropriately focusing on main results.

***Response 1:*** *Many thanks, we agree completely, and the discussion has been strengthened on page 27-28, lines 407-437. We have removed non statistically adjusted analysis in lines 478-484, 520-523, 528-535, 544-569) original version).*

**Comment 2:** Results, page 16 ".....In the adjusted multivariate Cox regression analysis indicated that intubated patients in ICU were 1.56 times at higher risk of death than those who were not intubated (aHR 1.56, 95%CI: 1.08-2.25, P = 0.017). Similarly, HIV-infected patients had 1.64 times increased risk of dying compared HIV negative patients (aHR 1.64, 95%CI: 1.11-2.36, P = 0.012). In contrast, myalgia on admission was associated with 32% reduced risk of dying compared to those who did not present with this clinical symptom (aHR 0.68, 95%CI: 0.47-0.97, P = 0.032) [Table 3a]." This sentence is repeated on page 17

***Response 2:*** *Thanks for this observation. On page 24, lines 363-369 (original version), this paragraph has been removed.*

**Comment 3:** same for statement " Furthermore, use of vancomycin,
enoxaparin, proton pump inhibitors (PPI), spironolactone, losartan and other
hypertensive drugs were associated with a low risk of mortality (aHR 0.62, 95%CI:
0.38-1.01, P = 0.011; aHR = 0.16, 95%CI: 0.06-0.46, P = 0.001; aHR 0.71, 95%CI:
0.53-0.97, P = 0.033; aHR = 0.50, 95%CI: 0.29-0.85, P = 0.011; aHR 0.58, 95%CI:
0.36-095, P = 0.03 and aHR 0.74, 95%CI: 0.55-0.99, P = 0.04 respectively) [Table 3b]." that is repeated on page 18
It would be good to see all drugs that were examined to have possible association in table 3

***Response 3:*** *Thanks for this comment. Amendment has been done accordingly. This amendment is on Page 25, lines 380-385. This paragraph has been removed (original document).*

**Comment 4:** It would be useful to report actual values of CRP, NT proBNP, CBC and not only HR

***Response 4:*** *Thanks. Page 18-19, Line 298-303, we have added actual values of CRP, NT proBNP, CBC in Table 2b.*

**Comment 5**: Were there any data on complications such as VAP, pulmonary embolism or pneumotorax?

***Response 5:*** *Thanks for this valuable concerns. On Page 31, lines 509-514, we have highlighted this point. “We did not record cases related to Ventilator-Associated Pneumonia, Pulmonary Embolism or Pneumothorax in our data. Many patients died suddenly, and as post-mortems were not performed on COVID-19 patients at the time, the cause of death (including suspected pulmonary embolism) could never be confirmed. This is unfortunately a limitation of this study.*

**Comment 6:** For Cox regression model-what was the follow up time? Fig 3 should be clearly labelled.

***Response 6:*** *Many thanks for the comment. Page 22-23, the follow up time was in days with longest patient staying more than 50 days in ICU. This has been corrected in Fig 2 and which has now been clearly labelled.*

**Comment 7:** What was the criteria for admission to ICU? Were there any patients on NIV? Was HFO available at the general ward/ high dependency unit as well?

***Response 7:*** *Thanks for identifying our missing point. During the first wave NIV and HFNO were not available in general wards and the high dependency unit was incorporated in ICU. The admission criteria and triage document are summarised in reference 28 and was strictly adhered to. On Page 8, line 167-170. We have added the following: “The initial assessment of the referred patient is focused on determining whether the patient is critically ill and requires ICU admission for ventilatory support or other organ support that is only available in the ICU [28].”.*

**Comment 8:** Mortality of intubated patients is likely related to severity of illness, or complications as authors noted, but time of intubation cannot be commented as it was not investigated.

***Response 8:*** *Thanks. On page 29, line 415-418, we have removed this statement (Original document).*

**Comment 9:** Please consider citing Risk Factors for Coronavirus Disease 2019 (COVID-19) Death in a Population Cohort Study from the Western Cape Province, South Africa. Clin Infect Dis. 2021 Oct 5;73(7):e2005-e2015. doi: 10.1093/cid/ciaa1198

***Response 9:*** *Thanks. this reference has been included in the discussion on the line 461-463.*

**Reviewer #2:**

The authors have presented important clinical data on COVID-19-related mortality in a large cohort of people (n=402) admitted to the intensive care unit (ICU) of a large teaching hospital in Cape Town, South Africa prior to COVID-19 vaccine availability. The authors provide important insights into factors contributing to COVID-19-related deaths in a middle-income setting with a relatively younger population and high burden of HIV compared to countries reporting similar data in the early days of the pandemic. Of particular interest is the fact that although people living with HIV (PLHIV) make up a small proportion of people requiring ICU admission for COVID-19 (14%), HIV remained a strong predictor of mortality in multivariable analysis 1.30 (1.06, 1.59).

**General comments:**

While the authors highlight the importance of the finding that HIV was a significant predictor of morality amongst ICU patients, the finding seems to get lost in the discussion owing to a lot of comparative data on crude results that are not generally in need of explanation.
As a reader, I am curious to know if any measures of CD4 count or viral load were taken or if at least an indication of ART-use (or is this a study limitation) to provide more insight into the relationship of HIV and COVID-19 mortality. Moreover, it would be important to highlight that although the common comorbidities leading to severe disease in high-income countries (diabetes, hypertension, and other cardiovascular diseases) were also common amongst patients admitted to the ICU in the present setting (despite being less prevalent in the general public), they were not significantly predictive of mortality when considered in the model (although asthma and increased BMI may be worth noting as trending toward significance). It would also be interesting to note (perhaps in an appendix) what comorbidities were most common among PLHIV in the cohort. It would also be helpful to note whether or not any patients admitted had active TB disease. In line 227 on page 10, the authors report 7% of people admitted had TB, but then refer to it as a “history of previous TB” in Table 1. If no one had active TB in ICU, this would be worth highlighting, although might there be a risk that concomitant TB went undetected given the focus on severe COVID-19 disease?

***Response:*** *Thank for this concern. Page 27-28 , line 407-435. We have clarified this concern as follows: “Several studies have found that the risk of ICU admission and death for COVID-19 among people living with HIV (PLWH) increased with age, consistent with an increased burden of comorbid conditions in older people [15, 35-38]. In contrast, another South African study found that while the proportion of PLWH was similar in surviving and deceased COVID-19 cases, a higher proportion of COVID-19 deaths occurred in patients aged 50 years or older in those living with HIV versus those who did not [39]. Diabetes (50%) and hypertension (42%) were present in a significant proportion of PLWH who died from COVID-19 [39]. The median (IQR) age of 56.7 (48.0-63.1) years was found to be associated with COVID-19 mortality in our study. This is consistent with findings in older people [35]. This study did not take into account important HIV confounders such as CD4 counts, viral load, and ART use. In COVID-19 infection, a lower CD4 cell count among PLWH was associated with increased mortality [40]. In the same line, a mortality odds ratio of 2.85 (95 percent CI 1.26–6.44) for CD4 cell count less than 350 cells/ml compared to higher values [39, 41] and higher mortality for CD4 cell count less than 200 cells/ml [39] were found. Similarly, the risk of COVID-19 hospitalization was 47 percent lower in people taking tenofovir disoproxil fumarate (TDF)/emtricitabine (FTC) (rate ratio 0.53, 95 percent CI 0.27–0.97) compared to people taking tenofovir alafenamide (TAF)/FTC [35, 42]. In analyses adjusted for baseline renal function, the hazard ratio of COVID-19 death for TDF/FTC compared to abacavir (ABC) or zidovudine (AZT) was 0.41 (95 percent CI 0.21–0.78) in South Africa [39]. An uncontrolled HIV viral load and/or a low CD4 count are directly related to a lack of recent or poor ART adherence. Tamuzi et al. demonstrated that SARS-CoV-2 could cause transient suppression of cellular immunity in PLWH, predisposing patients to exacerbated reactivation or new TB infection [23]. However, we did not collect data on active PTB in our study. Because only previous TB cases were reported, this could be explained by poor PTB screening among COVID-19 ICU patients”.*

**Specific comments:**

**Abstract:**

**Comment 1:** On page 3, lines 57-60, the authors mention sub-Saharan Africa’s lower COVID-19 case count, and potential explanations for it, but do not adequately come back to this point in the abstract’s conclusion or the manuscript’s discussion. I would suggest either excluding it from the abstract (and background) or returning to it in the discussion/conclusion based on what was observed in the study and bringing in NICD data on excess deaths in Western Cape province – it seems likely that case counts were better reported in Western Cape in this period, so under-reporting may be at play in other settings but less so in this one, and that lower prevalence of non-communicable diseases may also contribute.

***Response 1:*** *Thanks for this constructive comment. Line 57-60, 112-118. We have removed this paragraph from the abstract and background (original document).*

**Comment 2**: Page 3, line 74-75: “after adjusting for incident rate ratios” should read “after adjusting for confounding”

***Response 2****: Thanks, this has been corrected. Page 3, line 74. This is now read “after adjusting for confounding”.*

**Methods**

**Comment 3:** Page 9, line 192, the authors mention socioeconomic status as a sociodemographic variable. It would be helpful to know how this was measured (if indeed it was) and to report results by SES in tables 1 and 2.
It is not clear why age was dichotomized at the median, as age groups have been typically reported in other studies. Does the model still hold if age is dichotomized at >65 or as a continuous variable?

***Response 3:*** *Many thanks for the comment. Line 191-192, we have corrected as demographic and lifestyle characteristics. These include age, sex, smoking status and alcohol use.*

**Comment 4:** Please include a definition of low versus high pH under methods. This is important to be able to note if the statistically significant difference is also clinically significant.

***Response 4:*** *Many thanks, we agree completely, and information has been added on Page 9, line 195: “A pH above 7.45 was considered as an alkalemia”.*

**Findings:**

**Comment 5:** Please review tables and legends closely, as there are places where numbers and asterisks are not explained or comments between authors were included. It would also help to have clear cut points indicated for clinical parameters and arterial blood gasses in Table 3a. I believe from the interpretation of HRs that these are hazard ratios, not hazard rates.

***Response 5:*** *Thanks for this observation. This is well noted. We have made the change in Table 1, Table 2a, 2b, and 3a. This is seen in the lines 243-342*

**Comment 6:** Please note that the paragraph immediately preceding Table 3a is a repeat from page 16 (no line numbers are visible here), as are the paragraphs immediately preceding Table 3a and 3b.

***Response 6:*** *Thanks for the comment. Page 24-25, line 386-391, this paragraph has been removed. (Original document)*

**Discussion:**

**Comment 7:** It would help the reader to focus the discussion on the predictive factors of mortality and their clinical significance. As mentioned above, HIV is an important finding that needs more context.

***Response 7:*** *Thanks for the observation. We have broadly resolved this concern through the earlier response and adjustment made on. Page 27-28, line 407-435 in the general comment response.*

**Comment 8**: While it may seem obvious, it is also important to note that patients placed on mechanical ventilation were more likely sicker than those who were not, and it is unlikely that the use of mechanical ventilation led to mortality.

***Response 8:*** *Thanks for this comment. This Section has been added as per recommendation. Page 29, line 461-463: “Furthermore, increasing the occupancy of beds compatible with mechanical ventilation is associated with a higher mortality risk for ICU patients [45].”.*

**Comment 9:** The authors should also note under limitations that the number of comparisons made increases the likelihood of one or more spurious associations (we would expect 1/20 to be spurious with a significance of p=0.05).

***Response 9:*** *Thanks. Page 37, line 518-522. We have added the following: “the number of comparisons made increases the likelihood of one or more spurious associations”.*
