# Supplementary material for: Clinical characteristics associated with mortality of COVID-19 patients admitted to an intensive care unit of a tertiary hospital in South Africa
Source: PLoS One. 2022 Dec 30;17(12):e0279565. doi: 10.1371/journal.pone.0279565 (PMC9803161; doi:10.1371/journal.pone.0279565)
Supplement: Attachment — Submitted filename: plos-one.docx [file pone.0279565.s004.docx]

In this perspective, cohort study the authors reported the experience of ICU admission in patients with COVID-19 in a South African hospital, with the primary aim to assess the survival of these patients.

I understand this is a second-round revision, however, in my humble opinion, the manuscript continues to suffer major flaws that need to be considered by investigators. I agree with the previous reviewer who suggested applying more attention to the high prevalence of HIV infection in the South African population, which could give this manuscript uniqueness. However, the authors did not represent their own HIV patients with COVID-19. I believe the author needs to get more information about their own HIV patients.

As a reader of a manuscript coming from South Africa, I’ll be more interested to know about the South African variant of COVID-19. Unfortunately, the investigators did not address this critical issue in the current manuscript.

U Lalla et al reported the experience of South African hospital in ICU patients with COVID-19 (*Afr J Thorac Crit Care Med* 2021 Dec 31;27(4):10.7196/AJTCCM.2021.v27i4.185). The authors need to streamline why the current manuscript needs to be published as soon as there is a reported experience in critical ill COVID-19 patients from the same country.

No assessment of disease severity was provided. There is neither SOFA score nor APACHE score reported in the Tables. This is mandated in any study assessing outcomes of patients in ICU.

The manuscript had many writing errors in grammar and punctuation that need revision, as well as flaws that need to be considered by the authors:

**Introduction**

Line#108 the author reported many complications of severe COVID-19, among these, was encephalitis. In the cited reference [12] encephalitis was not among the reported complications. Please revise and consider adding VTE development from another reference.

**Methodology**

The planned recording of co-morbidities for the study did not include chronic renal failure, which appeared later in the result section. Please clarify in the methodology section all the recorded chronic illnesses.

Include either SOFA or APACHE score.

Line #192, the primary outcome of interest (of interest) appears to be redundant.

This is a prospective study. Please determine if the study patients were consented prior to enrollment, or if the investigators granted a consent waiver by the ethical body that cleared the study. This information needs to be addressed clearly in the methodology section.

**Results**

Tuberculosis could be abbreviated as TB as it appeared in the introduction section.

In the result section, line#231 the word days was unnecessarily repeated. This was observed in different parts of the result section. Please revise.

In Table.1 what was the aim of reporting the sample sizes related to each clinical characteristic? This need to be described for the reader. I suggest revising Table 1 in a way to facilitate reader understanding.

In line #245 the author reported the oxygenation in writing (less than 90) while it was reported as ( < ) at others. Please unify.

How was the PF ratio calculated in patients on admission to the hospital? Did all the patients undergo arterial line assessment on admission to the hospital? Some patients might be admitted with moderate COVID-19 and progress to severe during their hospital stay. Which PF ratio was entered for analysis?

How was the admission PF ratio statistically compared for those who died and those who survived? No statistical test was provided in the methodology section to perform head-to-head comparisons. The same applies to the comparison between those who were intubated in ICU versus before ICU admission (line#268). Please add to the statistical part section.

Table.2 For myalgia and DBP on univariate analysis show P value of 0.086. No P value was reported after adjustment ( P > 0.15). The same applies to variables reported under arterial blood pressure.

Many medications were selected for association testing. Which was the base to select these medications? (Table 3b). In the discussion section, anti-retroviral agents used to treat HIV were discussed without introducing this agent for association with COVID-19 outcomes. I believe as soon as this is a study in the area of the world with a high prevalence of AIDS, more focus needs to be directed toward medications used to manage HIV infection and to present them with analysis. The authors need to report in detail dexamethasone use with doses and duration.

In Table 3c the author reported eGFR. What was the method for assessing the eGFR. Nothing was mentioned in the methodology section.

In Table 3b, for the anticoagulants (enoxaparin) was that a therapeutic or prophylactic dose? This is crucial to report to know how many patients with indications for full anticoagulants were enrolled.

**Discussion**

The author continued to report the P values of the study in the discussion section. This should be transferred to the results section. ( for example lines 373 up to 375 on the first page of the discussion).

In the discussion section,

Line#376 extremely high is an overstatement. Please revise

Line#379, missing punctuation, and revising for the (% sign).

The study suffers many limitations. The authors need to add more of limitations in this study.
