# Supplementary material for: Clinical characteristics associated with mortality of COVID-19 patients admitted to an intensive care unit of a tertiary hospital in South Africa
Source: PLoS One. 2022 Dec 30;17(12):e0279565. doi: 10.1371/journal.pone.0279565 (PMC9803161; doi:10.1371/journal.pone.0279565)
Supplement: Attachment — Submitted filename: Reviewers_response 07_SEP_2022.docx [file pone.0279565.s005.docx]

**Reviewers ‘responses**

**Manuscript: Clinical characteristics associated with mortality of COVID-19 patients admitted to an Intensive Care Unit of a tertiary hospital in South Africa**

**Comment 1:** In this perspective, cohort study the authors reported the experience of ICU admission in patients with COVID-19 in a South African hospital, with the primary aim to assess the survival of these patients. I understand this is a second-round revision, however, in my humble opinion, the manuscript continues to suffer major flaws that need to be considered by investigators. I agree with the previous reviewer who suggested applying more attention to the high prevalence of HIV infection in the South African population, which could give this manuscript uniqueness. However, the authors did not represent their own HIV patients with COVID-19. I believe the author needs to get more information about their own HIV patients.

***Response 1:*** *Thank you for this valuable comment. In the manuscript with track changes line 300-302, we have included more information about the treatment of HIV-infected patients. “Among HIV-infected patients, only (13.9%) 54/388 were on ART and tenofovir disoproxil fumarate (TDF)/emtricitabine (FTC)/efavirenz (EFV) was the prevalent regimen (43.54%)”.*

**Comment 2:** As a reader of a manuscript coming from South Africa, I’ll be more interested to know about the South African variant of COVID-19. Unfortunately, the investigators did not address this critical issue in the current manuscript.

***Response 2:*** *Thanks for this comment. In the manuscript with track changes line 523-531, we have included the following: “SARS-CoV-2 changes over time due to the mutation in different viral proteins. Some mutations may affect the virus’s properties, such as how easily it spreads, the associated disease severity, or the performance of vaccines, therapeutic medicines, diagnostic tools, or other public health and social measures [55]. Those variants are named variants of concerns (VOCs).* *From the initial Wuhan variant, different VOCs have taken over South Africa, Alpha, Beta, Delta, and C.1.2 omicron and its different lineages are predominant in South Africa [55]. Compared with early variants of SARS-CoV-2 included in this study, new VOCs may be associated with an increase in the risk of ICU admission and death due to COVID-19.*

**Comment 3:** U Lalla et al reported the experience of South African hospital in ICU patients with COVID-19 (*Afr J Thorac Crit Care Med* 2021 Dec 31;27(4):10.7196/AJTCCM.2021.v27i4.185). The authors need to streamline why the current manuscript needs to be published as soon as there is a reported experience in critical ill COVID-19 patients from the same country. Lalla et al. compared the clinical characteristics, management, and outcomes of COVID-19 patients admitted to a South African ICU during the first and second waves. However, this manuscript focuses primarily on the first wave. In comparison to Lalla et al., this paper describes all epidemiological and clinical aspects of the first wave. This manuscript is significant because it was written at a time when there were few studies on COVID-19 patients admitted to the ICU in South Africa.

***Response 3:*** *Thank you for the valuable comment.*

**Comment 4:** No assessment of disease severity was provided. There is neither SOFA score nor APACHE score reported in the Tables. This is mandated in any study assessing outcomes of patients in ICU.

***Response 4:*** *Thanks for this comment, we did not gather data on the SOFA score or the APACHE score at the pick of the COVID-19 as case records did not have documented these scores.pa. This has been listed as the study’s limitation in the track change manuscript line 559-560.*

**Comment 5:** The manuscript had many writing errors in grammar and punctuation that need revision, as well as flaws that need to be considered by the authors:

***Response 5:*** *Thanks for this valuable comment. We have revised the grammar, punctuation, and flow throughout the manuscript.*

**Introduction**

**Comment 6:** Line#108 the author reported many complications of severe COVID-19, among these, was encephalitis. In the cited reference [12] encephalitis was not among the reported complications. Please revise and consider adding VTE development from another reference.

***Response 6:*** *Thanks. In the manuscript with track changes line 111, the reference related to encephalitis has been highlighted.*

**Methodology:**

**Comment 7:** The planned recording of co-morbidities for the study did not include chronic renal failure, which appeared later in the result section. Please clarify in the methodology section all the recorded chronic illnesses.

***Response 7:*** *Thanks. In the manuscript with track changes line 195, we have included: “chronic kidney disease (CKD)”.*

**Comment 8:** Include either SOFA or APACHE score.

***Response 8:*** *Thanks for this comment, we did not capture either the SOFA score or the APACHE score in our study. This has been listed as the study’s limitation line 559-560.*

**Comment 9:** Line #192, the primary outcome of interest (of interest) appears to be redundant.

***Response 9:*** *Thanks. We have corrected this in the manuscript with track, lines 198-199.*

**Comment 10:** This is a prospective study. Please determine if the study patients were consented prior to enrollment, or if the investigators granted a consent waiver by the ethical body that cleared the study. This information needs to be addressed clearly in the methodology section.

***Response 10:*** *Thanks. In the manuscript with track changes line 151, we have included the following: “**The ethical body that approved the study granted the investigators a consent waiver”.*

**Results**

**Comment 11:** Tuberculosis could be abbreviated as TB as it appeared in the introduction section.

***Response 11:*** *Thanks. “Tuberculosis” has been abbreviated as “TB” in the manuscript with track changes line 237.*

**Comment 12:** In the result section, line#231 the word days was unnecessarily repeated. This was observed in different parts of the result section. Please revise.

***Response 12:*** *Thanks. In the manuscript with track changes line 240, 274 the word “days” has been deleted.*

**Comment 13**: In Table.1 what was the aim of reporting the sample sizes related to each clinical characteristic? This need to be described for the reader. I suggest revising Table 1 in a way to facilitate reader understanding.

***Response 13:*** *Many thanks for the comment. The purpose of presenting the sample size was to show the number of observations for each characteristic since each characteristic did not have an equal sample size. We have included this statement at the bottom of Table 1. Furthermore, we have combined the last two columns into one column in Table 1 (Tracked manuscript pages 13 and 14).*

**Comment 14:** In line #245 the author reported the oxygenation in writing (less than 90) while it was reported as ( < ) at others. Please unify.

***Response 14:*** *Thanks. In the manuscript track changes line 265, we have harmonized this concern.*

**Comment 15:** How was the PF ratio calculated in patients on admission to the hospital? Did all the patients undergo arterial line assessment on admission to the hospital? Some patients might be admitted with moderate COVID-19 and progress to severe during their hospital stay. Which PF ratio was entered for analysis?

***Response 15:*** *Thanks for this valuable comment. In this manuscript, we have considered the initial PF at admission. All the patients admitted to the ICU were severe to critical COVID-19 cases.*

**Comment 16:** How was the admission PF ratio statistically compared for those who died and those who survived? No statistical test was provided in the methodology section to perform head-to-head comparisons. The same applies to the comparison between those who were intubated in ICU versus before ICU admission (line#268). Please add to the statistical part section.

***Response 16:*** *Thank you for the comment. We have updated the statistical methods section.*

*Chi-square and Wilcoxon ranksum tests were performed to test the population distribution associated with mortality among categorical variables and difference in medians for continuous variable with p-values. This statement has been added in statistical analysis section (Tracked manuscript lines 206-209).*

*As a result, the association of characteristics and the outcome are presented in Table 2a. In the current version, p-value is added for tests of association and equality of median (for example, admission PF ratio statistically compared for those who died and those who survived; the comparison between those who were intubated in ICU versus before ICU admission). Similarly, we have updated Table 2b (added p-value*: testing median equality of laboratory parameters compared with those who died and those who survived).*

**Comment 17:** Table.2 For myalgia and DBP on univariate analysis show P-value of 0.086. No P value was reported after adjustment (P > 0.15). The same applies to variables reported under arterial blood pressure.

***Response 17:*** *Thank you for the valuable comment. We have added all the characteristics that show p-value <0.15 in the unadjusted model in the final adjusted model in Table 2 (Tracked manuscript pages 16-18). As a result, Table 2a is updated in the manuscript (sore throat, myalgia, BP – diastolic also added in the adjusted model). However, there were correlations (multicollinearity issue) of pH with paCO2, K+, Lactate, HCO3std, So2c, and duration of pre-ICU admission; pf ratio with pao2, lactate, and so2c. As a result, we have excluded paCO2, paO2, Lactate, So2c, and duration of pre-ICU admission in an adjusted model due to multicollinearity even though the p-value is <0.15 in an unadjusted model. The above statement is added in Table 2a footnote in the manuscript. Table 3a is also updated in the manuscript since we have excluded So2c due to multicollinearity with pH even though the p-value is <0.15 in an unadjusted model. All results were also updated accordingly in the manuscript.*

*The summary of scatter matrix, correlation with a p-value of arterial blood gases parameter multicollinearity test is given below.*

*Variables with the number of observations less than 250 (rule of thumb) were not included in the adjusted model (e.g., AST in Table 2b).*

|  | ph | paco2 | pao2 | k | lactate | hco3std | so2c | pf_ratio | Duration of pre-ICU admission | BP – Diastolic |
| --- | --- | --- | --- | --- | --- | --- | --- | --- | --- | --- |
| ph | 1 |  |  |  |  |  |  |  |  |  |
| paco2 | -0.386 | 1 |  |  |  |  |  |  |  |  |
| p-value | 0.000 |  |  |  |  |  |  |  |  |  |
| pao2 | -0.023 | -0.059 | 1 |  |  |  |  |  |  |  |
| p-value | 0.646 | 0.244 |  |  |  |  |  |  |  |  |
| k | -0.448 | 0.174 | -0.021 | 1 |  |  |  |  |  |  |
| p-value | 0.000 | 0.001 | 0.676 |  |  |  |  |  |  |  |
| lactate | -0.510 | 0.097 | 0.003 | 0.190 | 1 |  |  |  |  |  |
| p-value | 0.000 | 0.056 | 0.949 | 0.000 |  |  |  |  |  |  |
| hco3std | 0.228 | 0.064 | 0.070 | -0.044 | -0.151 | 1 |  |  |  |  |
| p-value | 0.000 | 0.219 | 0.176 | 0.402 | 0.004 |  |  |  |  |  |
| so2c | 0.272 | -0.132 | 0.398 | -0.118 | -0.321 | 0.076 | 1 |  |  |  |
| p-value | 0.000 | 0.009 | 0.000 | 0.021 | 0.000 | 0.149 |  |  |  |  |
| pf_ratio | -0.081 | -0.109 | 0.717 | -0.037 | 0.154 | 0.035 | 0.365 | 1 |  |  |
|  | 0.111 | 0.030 | 0.000 | 0.464 | 0.003 | 0.505 | 0.000 |  |  |  |
| Duration of pre-ICU admission | 0.138 | -0.056 | 0.049 | -0.037 | -0.129 | 0.032 | 0.094 | 0.173 | 1 |  |
| p-value | 0.006 | 0.264 | 0.334 | 0.468 | 0.011 | 0.540 | 0.065 | 0.001 |  |  |
| BP – Diastolic | 0.075 | -0.177 | 0.021 | 0.029 | -0.034 | -0.003 | 0.022 | 0.084 | 0.119 | 1 |
| p-value | 0.141 | 0.001 | 0.683 | 0.569 | 0.517 | 0.950 | 0.673 | 0.104 | 0.019 |  |

**Comment 18:** Many medications were selected for association testing. Which was the base to select these medications? (Table 3b). In the discussion section, anti-retroviral agents used to treat HIV were discussed without introducing this agent for association with COVID-19 outcomes. I believe as soon as this is a study in the area of the world with a high prevalence of AIDS, more focus needs to be directed toward medications used to manage HIV infection and to present them with analysis. The authors need to report in detail dexamethasone use with doses and duration.

***Response 18:*** *Thank you for this comment. In the manuscript with track changes page 27, we had only the medications listed and added the corticosteroids in Table 3b and line 346-349. In the tracked manuscript line 300-302, we have included the following the rate of ART among PLWH: “However, among HIV-infected patients, only (13.9%) 54/388 were on ART and TDF/FTC/EFV was the main regimen (43.54%)”.*

**Comment 19:** In Table 3c the author reported eGFR. What was the method for assessing the eGFR. Nothing was mentioned in the methodology section.

***Response 19:*** *Thanks. In the manuscript with track changes line 195, we have added in the methodology section (in outcomes and predictor variables section) as a normal eGFR is 60 or more.*

**Comment 20:** In Table 3b, for the anticoagulants (enoxaparin) was that a therapeutic or prophylactic dose? This is crucial to report to know how many patients with indications for full anticoagulants were enrolled.

***Response 20:*** *Thanks for this comment. In table 3b, page 27, we have specified that enoxaparin was given as a prophylactic dose. This means that enoxaparin was administered to all the COVID-19 patients admitted to the ICU.*

**Discussion**

**Comment 21:** The author continued to report the P values of the study in the discussion section. This should be transferred to the results section (for example lines 373 up to 375 on the first page of the discussion).

***Response 21:*** *Thanks. In the manuscript with track changes lines 405, 419, 432, 434, 467, 468, 475, 479, 481 and 483, we have corrected this.*

**Comment 22:** Line#376 extremely high is an overstatement. Please revise

***Response 22:*** *Thanks. In the manuscript with track changes, line 408, we have removed the adverb “extremely”*

**Comment 23:** Line#379, missing punctuation, and revising for the (% sign).

***Response 23:*** *Thanks. In the manuscript with track changes lines 450, 453, and 455, the change has been made.*

**Comment 24:** The study suffers many limitations. The authors need to add more limitations to this study.

***Response 24:*** *Thanks. In the manuscript with track changes, lines 559-560, we have included the following: “The study did not capture either the SOFA or the APACHE scores”.*
