# Supplementary material for: Clinical characteristics associated with mortality of COVID-19 patients admitted to an intensive care unit of a tertiary hospital in South Africa
Source: PLoS One. 2022 Dec 30;17(12):e0279565. doi: 10.1371/journal.pone.0279565 (PMC9803161; doi:10.1371/journal.pone.0279565)
Supplement: Attachment — Submitted filename: Reviewers_Response_05_Dec_2022.docx [file pone.0279565.s006.docx]

**Reviewers ‘responses**

**Manuscript: Clinical characteristics associated with mortality of COVID-19 patients admitted to an Intensive Care Unit of a tertiary hospital in South Africa**

**Comment 1:** Abstract, Line 70 (Of 402 patients…etc) please rephrase.

***Response 1:*** *Thanks. In the tracking manuscript line 71-73, we have rephrased as follows: “Of the 402 patients admitted to the ICU, 250 (62%) died, and another 12 (3%) died in the hospital after being discharged from the ICU”.*

**Comment 2:** Line 95, I would like you to add at least a sentence specifying for example that susceptibility to SARS-CoV-2 is universal, but older age have always been associated with disease severity (and high mortality), add this citation to support your statement:
*<https://doi.org/10.3390/healthcare10071341>

***Response 2:*** *Thank you for this substantial input. In the tracking manuscript, line 96-98, we have included this: “Although SARS-CoV-2 susceptibility is universal, older age has always been associated with disease severity (and high mortality) [4]”.*

**Comment 3**: Line 96, please update data.

***Response 3:*** *Thank you for making this observation. We have updated the COVID-19 data in the tracking manuscript lines 99-101 to include data from November 7, 2022.*

**Comment 4:** Line 112, COVID-19 instead of Covid-19

***Response 4:*** *Thanks. We have corrected this as highlighted in the tracking manuscript line 115*

**Comment 5:** Line 142, please add rheumatic diseases with diabetes and hypertension and add these citations:
*<https://doi.org/10.1001/jama.2020.12839>
*<https://doi.org/10.1016/j.nmni.2021.100846>

***Response 5:*** *Thanks. These two references are included in the tracking manuscript on line 147. It now reads: “however, there is little known about clinical outcomes of COVID-19 patients with HIV, TB, and post-TB lung disease (PTLD), rheumatic diseases, diabetes and hypertension [26, 27]”.*

**Comment 6**: Line 164, COVID-19 again, please correct it in the whole document.

***Response 6:*** *Thank you; this has been corrected in tracking manuscript line 167.*

**Comment 7:** Your results section is much more improved compared to the first submission, congratulations for that.

***Response 7:*** *Thank you so much for this appreciation.*

**Comment 8:** The fact that this study was conducted in 2020, there were no vaccines at that moment, this can be considered as a limitation that should be added in your limitations section.

***Response 8:*** *Thanks for this. In the tracking manuscript line 557-559, we have included the following: “Since the data were collected prior to COVID-19 vaccination, vaccination-related information was not included”.*

**Comment 9:** I suggest adding more results (numbers) in your conclusion.

***Response 9:*** *Thanks. We included the results as numbers in the conclusion of the tracking manuscript lines 564, 568-570.*

**Comment 10:** I also suggest to the authors to make another English revision, I know that it’s the second revised version, but it’s better to carefully check the manuscript again.

***Response 10:*** *Thank you for your helpful feedback; we have revised the English throughout the manuscript.*
